# Circular RNA circ-MMP11 Contributes to Lapatinib Resistance of Breast Cancer Cells by Regulating the miR-153-3p/ANLN Axis

**DOI:** 10.3389/fonc.2021.639961

**Published:** 2021-07-06

**Authors:** Xiaoli Wu, Yi Ren, Rong Yao, Leilei Zhou, Ruihua Fan

**Affiliations:** ^1^ Department of Pharmacy, The Affiliated Huaian No.1 People’s Hospital of Nanjing Medical University, Huaian, China; ^2^ Department of Thyroid and Mammary Gland, The Affiliated Huaian No.1 People’s Hospital of Nanjing Medical University, Huaian, China; ^3^ Department of Oncology, The Affiliated Huaian No.1 People’s Hospital of Nanjing Medical University, Huaian, China

**Keywords:** circ-MMP11, miR-153-3p, ANLN, breast cancer, lapatinib

## Abstract

**Background:**

Drug-resistance is a major obstacle to the treatment of breast cancer. Circular RNA (circRNA) circ-MMP11 has been reported to be promoting the progression of breast cancer. This study is designed to explore the role and mechanism of circ-MMP11 in lapatinib resistance in breast cancer.

**Methods:**

Circ-MMP11, microRNA-153-3p (miR-153-3p), and Anillin (ANLN) levels were detected by real-time quantitative polymerase chain reaction (RT-qPCR). Cell viability, number of colonies, apoptosis, migration, and invasion were detected by 3-(4,5-dimethyl-2-thiazolyl)-2,5-diphenyl-2-H-tetrazolium bromide (MTT), colony formation, flow cytometry, and transwell assays, respectively. Exosomes were exerted and detected by differential centrifugation and a transmission electron microscope. The protein levels of CD63, CD9, and ANLN were assessed by western blot assay. The binding relationship between miR-153-3p and circ-MMP11 or ANLN was predicted by circinteractome or starbase, and then verified by a dual-luciferase reporter assay and RNA pull-down assay. The biological role of circ-MMP11 on breast cancer tumor growth and drug resistance was detected by the xenograft tumor model *in vivo.*

**Results:**

Circ-MMP11 and ANLN were highly expressed, and miR-153-3p was decreased in LR breast cancer tissues and cells. Circ-MMP11 could be transported by exosomes. Furthermore, circ-MMP11 knockdown promoted lapatinib sensitivity by repressing cell viability, colony number, migration, invasion, and boosting apoptosis in LR breast cancer cells. Circ-MMP11 deficiency improved the drug sensitivity of breast cancer *in vivo.* Mechanically, circ-MMP11 could regulate ANLN expression through sponging miR-153-3p.

**Conclusion:**

Circ-MMP11 could be transferred by exosomes in breast cancer cells. And circ-MMP11 functioned as a sponge of miR-153-3p to regulate ANLN expression, thereby promoting lapatinib resistance in breast cancer cells, providing therapeutic targets for the treatment of breast cancer.

## Introduction

Like malignant tumors, breast cancer has caused a serious burden on the patient’s quality of life and psychological state (1). According to cancer statistics by 2020, there is approximately 30% breast cancer in females diagnosed cancer in the United States, especially among younger women aged 20 to 59 years (2). The early detection of breast cancer is relatively difficult in China, most patients usually are diagnosed at an advanced stage with a poor prognosis. Notably, lapatinib, an oral small-molecule tyrosine kinase inhibitor, has greatly improved the clinical benefits due to the high efficiency and low side effects (3, 4). Unfortunately, like other molecular targeting drugs, innate or acquired lapatinib resistance is becoming somewhat compromised for the treatment of human cancer clinically (5–7). Thus, to identify effective therapeutic targets to increase the lapatinib sensitivity in breast cancer, it is necessary to explore the molecular mechanism of drug resistance.

In recent decades, circular RNAs (circRNAs), a class of non-coding transcripts, have attracted extensive attention owing to their unique closed-loop structure (8). Mounting evidence has suggested that circRNAs were implicated in the formation and development of human tumors, serving as oncogenes or tumor suppressors. Furthermore, it was previously documented the involvement of dysregulated circRNAs in the development of drug-resistance in breast cancer (9). The forced expression of circ-RNF111 promoted paclitaxel resistance, cell growth, metastasis, and glycolysis through the miR-140-5p/E2F3 axis in breast cancer (10). Also, circRNA_0025202 weakened the malignancy and improved the tamoxifen sensitivity of breast cancer cells by sponging miR-182-5p (11). In a recent report, circ-MMP11 (hsa_circ_0062558) functioned as a competitive endogenous RNA (ceRNA) of miR-1204 to accelerate the progression of breast cancer (12). Nevertheless, little is known about the value of circ-MMP11 in drug-resistance and progression of breast cancer.

A group of 40–100 nm membrane vesicles, exosomes have been verified to be activity released into the extracellular environment by tumor cells (13). It is becoming increasingly apparent that tumor-derived exosomes are correlated with metastasis and drug resistance in different cancers (14). Some literature has shown that circRNAs widely exist in exosomes, which suggests the clinical significance of exosomal circRNA in the diagnosis and prognosis of human tumors (15). Actually, several reports have demonstrated that exosomal circRNAs played a vital role in tumor progression and drug-resistance in human cancers (16, 17). To our knowledge, whether circ-MMP11 can be transferred by exosomes is still unknown.

In this study, our data showed that circ-MMP11 was upregulated in the lapatinib-resistant (LR) breast cancer tissues and cells. Furthermore, these findings suggested that circ-MMP11 could be mediated transfer by exosomes, and circ-MMP11 could enhance the lapatinib resistance through regulating the miR-153-3p/Anillin (ANLN) in breast cancer.

## Materials and Methods

### Clinical Samples and Cell Culture

According to the response to the advanced breast cancer after treatment with lapatinib based molecular targeted therapy from the breast clinic of The Affilicated Huaian No.1 People’s Hospital of Nanjing Medical University were divided into two groups: 27 drug-resistant patients and 21 drug-sensitive patients followed by the collection of tissues samples. Written informed consent was signed from each participant. Also our experiment was approved by the Ethics Committee of The Affiliated Huaian No.1 People’s Hospital of Nanjing Medical University.

Normal human mammary epithelial cell line (MCF-10A: ATCC^®^ CCL-10317), and human breast cancer cell lines (MDA-MB-231: ATCC^®^ HTB-26 and MCF-7: ATCC^®^ HTB-22) were provided by the American Type Culture Collection (ATCC, Manassas, VA, USA). At 37°C with an atmosphere of 5% CO_2_ in the incubator, cells were grown in Dulbecco’s modified Eagle’s medium (DMEM; Gibco, Rockville, MD, USA) with 10% fetal bovine serum (FBS; Invitrogen, Carlsbad, CA, USA). In addition, LR breast cancer cell lines (MDA-MB-231/LR and MCF-7/LR cells) were established from parental cells through gradual exposure to increasing lapatinib (Sigma-Aldrich, St. Louis, MO, USA) concentrations (for 5 to 250 nM) as previously described (18).

### Real-Time Quantitative Polymerase Chain Reaction (RT-qPCR)

Based on the instruction of TRIzol reagent (Invitrogen), total RNA from tissues and cells were generated, followed by synthesis for cDNA with a PrimeScript^™^ RT Master Mix (TaKaRa, Tokyo, Japan). And then, on a 7500 Real-Time PCR System (Applied Biosystems, Foster City, CA, USA), qPCR analysis was performed with a SYBR Green PCR kit (TaKaRa). The glyceraldehyde-3-phosphate dehydrogenase (GAPDH) was acted as an endogenous control for circ-MMP11, linear MMP11, and ANLN, whereas U6 was used for miR-153-3p. Then, the 2^–ΔΔCt^ method was employed for the calculation of relative RNA expression. The primers in this study were presented in **Table 1**.

**Table 1:** The sequences of primers for RT−qPCR used in this study.

Names	Sequences (5’-3’)
Circ-MMP11: Forward	CTAGCTATGCCTACTTCCTGCG
Circ-MMP11: Reverse	CCAGAGCCTTCACCTTCACA
Linear MMP11: Forward	GCAGGGACTACTGGCGTTTC
Linear MMP11: Reverse	CGCGCAGGAAGTAGGCATAG
MiR-153-3p: Forward	TTGCATAGTCACAAAAGTGAT
MiR-153-3p: Reverse	CAGTGCGTGTCGTGG AGT
ANLN: Forward	CAGACAGTTCCATCCAAGGGAG
ANLN: Reverse	CTTGACAACGCTCTCCAAAGCG
U6: Forward	CTCGCTTCGGCAGCACA
U6: Reverse	AACGCTTCACGAATTTGCGT
GAPDH: Forward	GTGGACCTGACCTGCCGTCT
GAPDH: Reverse	GGAGGAGTGGGTGTCGCTGT

### Drug Resistance Assay

In this assay, lapatinib resistance and cell viability were detected by 3-(4, 5-dimethyl-2-thiazolyl)-2, 5-diphenyl-2-H-tetrazolium bromide (MTT, Sigma-Aldrich) assay. For the analysis of lapatinib resistance, un-transfected or transfected cells were treated with different doses of lapatinib (0, 4, 8, 12, 16, 20, 24, 28, and 32 nM) for 48 h. Subsequently, at the specified time points, 20 μl MTT solution (5 mg/ml, Sigma-Aldrich) was added into each well for another 4 h, and then, 150 μl dimethyl sulfoxide (DMSO, Sigma-Aldrich) was added for dissolution for formed formazan crystals. At last, a microplate reader (Bio-Tek Instruments, Hopkinton, MA, USA) was used for measurement of cell absorbance at 490 nm, and the relative survival curve shown the concentration of lapatinib causing 50% inhibition of growth (IC_50_). For detection of cell viability, transfected cells were conducted using MTT assay at the indicated time points.

### Ribonuclease R (RNase R) and Subcellular Fractionation Assay

For RNase R digestion, RNase R (3 U/mg, Epicentre, San Diego, California, USA) was applied for the treatment of total RNA at 37°C, followed by incubation for 15 min. After purification, the samples were subject to RT-qPCR analysis of circ-MMP11 and linear MMP11 levels.

For subcellular fractionation assay, MDA-MB-231/LR and MCF-7/LR cells were suspended in cytoplasm lysis buffer, followed by centrifugation for the separation of cell fractions. Then, the supernatant was transferred to fresh RNase-free tubes, while nucleus lysis buffer was added into other fresh RNase-free tubes with the pellet. After extraction of cytoplasmic and nuclear RNA with TRIzol regent (Invitrogen), RT-qPCR assay was applied to detect the expression of circ-MMP11, U6 (nucleus control), and GAPDH (cytoplasm control), respectively.

### Exosome Detection and Treatment

For exosome detection, exosomes from LR breast cancer cells in line with the operation manual of ultracentrifugation and total exosome isolation kit (Invitrogen). Subsequently, according to the previously described (19), a transmission electron microscope (HT7700, Hitachi, Tokyo, Japan) was carried out for the examination of extracted exosomes.

For inhibition of exosome generation, LR cells were treated with or untreated the inhibitor of exosome release GW4869 (Sigma-Aldrich) at 10 μM concentration for 48 h. Then, the expression level of circ-MMP11 in the culture supernatants was detected using RT-qPCR assay.

### Western Blot Assay

After the extraction of total proteins from exosomes or cells using RIPA buffer (Beyotime, Nantong, China), the treated samples (50 μg) were separated *via* 10% SDS-PAGE and moved to nitrocellulose membranes (Millipore, Bedford, MA, USA), followed by blockage with 5% nonfat milk for 1 h. Then, the membranes were incubated with primary antibodies CD63 (1:1,000, ab216130, Abcam, Cambridge, MA, USA), CD9 (1:1,000, ab92726, Abcam), ANLN (1:1,000, ab211872, Abcam), and GAPDH (1:1,000, ab9485, Abcam) at 4°C overnight, which were further probed with the secondary antibody: horseradish peroxidase-conjugated goat-anti-rabbit (ab205178, 1:10,000, Abcam) for 1h. As per the guide book of the ECL detection kit (GE Healthcare, Piscataway, NJ, USA), the bands were detected in this study.

### Cell Transfection

Circ-MMP11 small interfering RNA (si-circ-MMP11, 5’-ACACAGTTGTTTTCTAGCTAT-3’), si-ANLN (5’-GCAAACAACTAGAAACCAATT-3’), miR-153-3p mimic (miR-153-3p, 5’-TTGCATAGTCACAAAAGTGATC-3’), miR-153-3p inhibitor (anti-miR-153-3p, 5’-GATCACTTTTGTGACTATGCAA-3’), and their negative controls (si-NC, miR-NC, and anti-miR-NC) were acquired from Ribobio (Guangzhou, China). Meanwhile, circ-MMP11 or ANLN overexpression vector was constructed by the introduction of the sequence of circ-MMP11 or ANLN into pcDNA3.1 vector (Invitrogen), namely pcDNA3.1-circ-MMP11 (circ-MMP11) or pcDNA3.1-ANLN (ANLN), and pcDNA3.1 empty vector (Invitrogen) worked as their controls (vector, pcDNA). With the help of Lipofectamine 3000 (Invitrogen), all transfection of MDA-MB-231/LR and MCF-7/LR cells was conducted. After incubation for 48 h, cells were acquired for subsequent experiments.

### Colony Formation Assay

Transfected MDA-MB-231/LR and MCF-7/LR cells (5 × 10^2^ cells/well) were introduced into six-well plates, followed by incubation for 2 weeks at 37°C. After discarding the medium, these cells were stained with 0.1% crystal violet (Sigma-Aldrich) after being fixed in 4% paraformaldehyde for 30 min. At last, visible colonies were imaged with microscopy (Nikon, Tokyo, Japan), followed by calculation with Image J (NIH, Bethesda, MD, USA).

### Cell Apoptosis Assay

In the assay, transfected MDA-MB-231/LR and MCF-7/LR cells were harvested, followed by fixation with ethanol (Sigma-Aldrich) for 2 h. After re-resuspended in Binding Buffer, treated cells were stained with 5 μl Annexin (V-fluorescein isothiocyanate) V-FITC (Selleck, Shanghai, China) and 10 μl Propidium Iodide (PI) (Selleck) in the dark for 10 min. Then, a FACSan flow cytometry (BD Bioscience, San Jose, CA, USA) was used for the detection of apoptosis rates.

### Transwell Assays

For migration assay, transfected MDA-MB-231/LR and MCF-7/LR cells at the density of 5 × 10^4^ cells/well were added to the upper chamber (Costar, Cambridge, Massachusetts, USA) with serum-free culture medium, followed by addition with the medium containing 10% FBS (Invitrogen) into the bottom counterpart. Some 24 h later, the cells attached to the bottom surface were stained with 0.1% crystal violet after being fixed methanol for 30 min. Then, the migrated cells were analyzed with an inverted microscope (Tecan, Switzerland, magnification ×100). For invasion assay, cells (1 × 10^6^ cells/well) were plated in the upper chamber (Costar) of matrigel-coated inserts (BD Bioscience, San Jose, CA, USA), and the subsequent test procedure was consistent with the migration assay.

### Dual-Luciferase Reporter Assay

The underlying binding sites between miR-153-3p and circ-MMP11 or ANLN 3’UTR were predicated using circinteractome or Starbase software and the following dual-luciferase reporter assay verified the prediction. In brief, the sequences of circ-MMP11 and ANLN 3’UTR possessing wild-type (WT) or the site-directed mutant-type (MUT) (QuikChange Site-Directed Mutagenesis kit, Stratagene, La Jolla, CA, USA) miR-153-3p binding sites were amplified and cloned into pmirGLO plasmids (Promega, Madison, WI, USA). Whereafter, referring to the instruction guidelines of Lipofectamine 3000 (Invitrogen), the constructed reporter vectors and miR-153-3p or miR-NC were co-transfected into MDA-MB-231/LR and MCF-7/LR cells. After incubation for 48 h, the assessment of firefly and renilla luciferase activity was performed using a dual-luciferase reporter assay system (Promega).

### RNA Pull-Down Assay

According to the producer’s instructions of Lipofectamine RNAiMax Reagent (Invitrogen), biotin-labeled miR-153-3p or miR-NC (bio-miR-153-3p or bio-miR-NC) were transfected into MDA-MB-231/LR and MCF-7/LR cells. After harvesting and sonicating, the cell lysates were treated with streptavidin agarose beads (Invitrogen) for 2 h to pull down the biotin-coupled RNA complex. Finally, the RNeasy Mini Kit (Qiagen, Duesseldorf, Germany) was applied to refine the samples, which then were further analyzed using RT-qPCR assay.

### Tumor Xenograft Assay

This experiment got the approval of the Animal Ethics Committee of The Affiliated Huaian No.1 People’s Hospital of Nanjing Medical University. First of all, GeneChem (Shanghai, China) provided a stable circ-MMP11 knockdown vector: lentiviral-based short hairpin RNA (shRNA) targeting circ-MMP11 (sh-circ-MMP11) and negative control (sh-NC). Then, female BALB/C nude mice (Vital River Laboratory, Beijing, China), aged 3–4 weeks old, were kept in a specific pathogen-free environment, and divided into four groups (the sh-NC group, the sh-NC + Lapatinib group, the sh-circ-MMP11 group, and the sh-circ-MMP11 + Lapatinib group). The mice (n = eight per group) were subcutaneously injected the right flank with MDA-MB-231/LR cells (6 × 10^6^) with sh-circ-MMP11 or sh-NC, and intraperitoneally injected with Lapatinib. Eight days after the injection, the mice were administrated with 20 mg/kg lapatinib (the sh-NC + Lapatinib group and the sh-circ-MMP11 + Lapatinib group) or the same amount of PBS (the sh-NC group and the sh-circ-MMP11 group) every three days. Tumor volume was measured at the indicated time points (8, 11, 14, 17, 21, and 23 days). Some 23 days after treatments, the tumors were obtained, followed by a photograph, weight, and analysis with RT-qPCR assay.

### Statistical Analysis

All statistical analyses in this study were carried out using GraphPad Prism7 software. Between two and multiple groups were compared with Student’s *t*-test or one-way analysis of variance (ANOVA) with Tukey’s tests. The assessment data were exhibited as the mean ± standard deviation (SD). Pearson correlation analysis was applied for expression correlation between miR-153-3p and circ-MMP11 or ANLN. A *P*-value of less than 0.05 was deemed as statistically significant.

## Results

### Circ-MMP11 Expression Was Upregulated in LR Breast Cancer Tissues and Cells

**Figure 1:**
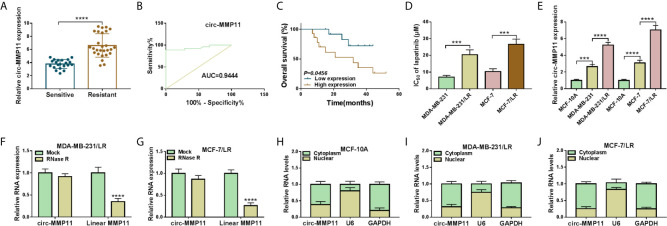
Expression patterns of circ-MMP11 in LR breast cancer tissues and cells. **(A)** RT-qPCR assay was applied to assess the expression level of circ-MMP11 in the drug-sensitive group (n = 21) and the drug-resistant group (n = 27). **(B)** ROC curve analysis for the diagnostic value evaluation of circ-MMP11 in breast cancer patients. **(C)** The overall survival of breast cancer patients with circ-MMP11 high or low level was determined by Kaplan–Meier analysis. **(D)** MTT assay was used to analyze the IC_50_ value of lapatinib in breast cancer cell lines (MDA-MB-231 and MCF-7), and LR-resistant breast cancer cell lines (MDA-MB-231/LR and MCF-7/LR cells). **(E)** Relative circ-MMP11 expression was detected in MCF-10A, MDA-MB-231, MDA-MB-231/LR, MCF-7, MCF-7/LR cells. **(F, G)** Expression levels of circ-MMP11 and linear MMP11 were examined in MDA-MB-231/LR and MCF-7/LR cells treated with RNase R or Mock. **(H–J)** The cellular localization of circ-MMP11 in MCF-10A cells and LR breast cancer cells was analyzed by Subcellular fractionation assay. ***P < 0.001, ****P < 0.0001 (Student’s *t*-test or ANOVA with Tukey’s tests), circ-MMP11 relative to GAPDH.

First of all, to investigate the role of circ-MMP11 on lapatinib resistance in breast cancer, its expression level was detected by RT-qPCR assay. As presented in **Figure 1A**, circ-MMP11 level was increased in the drug-resistant group (n = 27) compared with the drug-sensitive group (n = 21). Meanwhile, ROC curve analysis was conducted to assess the potential of circ-MMP11 as a diagnostic marker for breast cancer patients. As presented **Figure 1C**, the ROC curve data suggested that the AUC reached 0.9444 in circ-MMP11 (**Figure 1B**). Based on Kaplan–Meier analysis, the LR breast cancer patients in the low circ-MMP11 level group had a higher survival rate than those in the high circ-MMP11 level group (**Figure 1C**). Next, we further explored the lapatinib resistance in breast cancer cells. In this assay, cells were first treated with different concentrations of lapatinib for 48 h, followed by an assessment of IC_50_ value through MTT assay. According to the data shown in **Figure 1D**, IC_50_ value of lapatinib in MDA-MB-231/LR and MCF-7/LR cells was significantly higher than that in MDA-MB-231 and MCF-7 cells, suggesting the production of LR resistance in MDA-MB-231/LR and MCF-7/LR cells. Moreover, we further verified that circ-MMP11 was apparently upregulated in MDA-MB-231/LR and MCF-7/LR cells in comparison with normal human mammary epithelial cell line (MCF-10A) and their parental cell lines (MDA-MB-231 and MCF-7) (**Figure 1E**). Then, to detect the stability of circ-MMP11, MDA-MB-231/LR and MCF-7/LR cells were treated with RNase R. As displayed in **Figures 1F, G**, the treatment of RNase R decreased the RNA level of Linear MMP11, while had no evident effect on circ-MMP11. Besides, the subcellular fractionation assay validated the cytoplasmic localization of circ-MMP11 in MCF-10A cells (**Figure 1H**). Meanwhile, circ-MMP11 was also predominantly located in the cytoplasm of MDA-MB-231/LR and MCF-7/LR cells (**Figures 1I, J**), manifesting the underlying post-transcriptional regulatory mechanism of circ-MMP11 in LR breast cancer cells.

### Circ-MMP11 Was Transported by Exosomes

**Figure 2:**
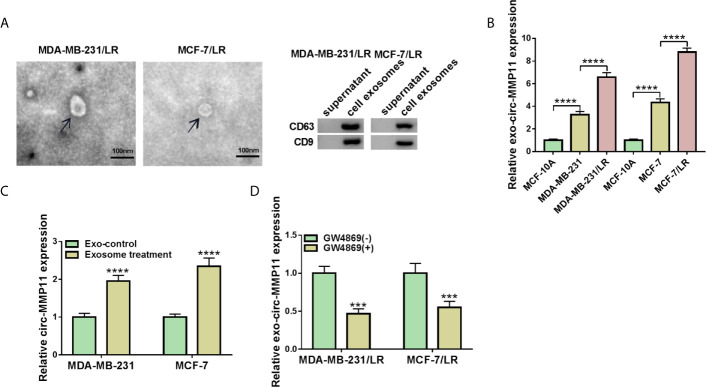
Exosomal circ-MMP11 was increased in LR breast cancer cells. **(A)** Transmission electron microscope was employed to analyze the exosome from the cell culture medium of MDA-MB-231/LR and MCF-7/LR cells. And exosomal markers CD63 and CD9 were detected by western blot assay. **(B)** Circ-MMP11 level was measured in MCF-10A, MDA-MB-231, MDA-MB-231/LR, MCF-7, MCF-7/LR cells. **(C)** Relative circ-MMP11 expression was assessed in MDA-MB-231 and MCF-7 cells treated with control or exosome. **(D)** Circ-MMP11 level was tested in the corresponding cultured media of MDA-MB-231/LR and MCF-7/LR cells treated with or without GW4869. ***P < 0.001, ****P < 0.0001 (ANOVA with Tukey’s tests), circ-MMP11 relative to GAPDH.

Then, we further explored whether circ-MMP11 could be transported by the exosome. To begin with, exosome were extracted from MDA-MB-231/LR and MCF-7/LR cells, followed by the detection of exosomes morphology using the transmission electron microscope. As presented in **Figure 2A**, the diameter of exosomes was about 100 nm, which was a typical rounded particle. Also, western blot assay demonstrated the expression of exosomal marker protein CD63 and CD9 in LR cell lines relative to the parental cell lines (**Figures 2A** and **S1**). Of note, we found that the level of exo-circ-MMP11 derived from drug-resistant cells was also higher than that of both drug-sensitive cells and MCF-10A cells (**Figure 2B**). Next, to verify whether circ-MMP11 could be delivered by exosomes, exosomes from resistant cells or MCF-10A cells were co-incubated with MDA-MB-231 and MCF-7 cells. Data suggested that circ-MMP11 level was increased in cells treated with exosomes from resistant cells relative to cells with exosomes from MCF-10A cells (**Figure 2C**). Also, we applied GW4869, a known blocker for exosomes, to inhibit the exosome secretion. And we noticed that the expression level of exo-circ-MMP11 was lower in cultured media of MDA-MB-231/LR and MCF-7/LR cells treated with GW4869 than that in cultured media of cells without GW4869 (**Figure 2D**). Besides, our data suggested that GW4869 treatment decreased miR-153-3p and ANLN level and cell proliferation ability (**Figure S2**). All of the results suggested that circ-MMP11 could be transported by exosomes.

### Circ-MMP11 Knockdown Enhanced Lapatinib Sensitivity in LR Breast Cancer Cells

**Figure 3:**
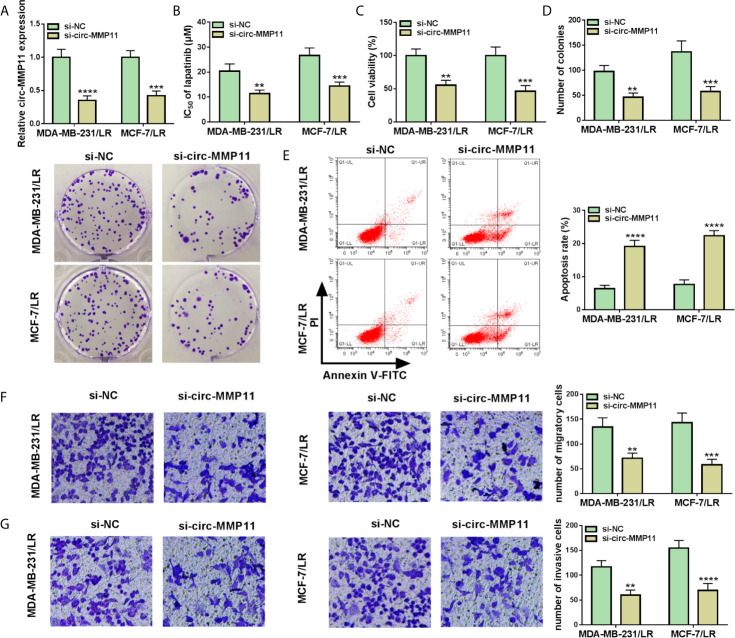
Circ-MMP11 knockdown decreased lapatinib resistance in LR breast cancer cells. MDA-MB-231/LR and MCF-7/LR cells were transfected with si-NC and si-circ-MMP11**. (A)** Expression level of circ-MMP11 was detected in transfected MDA-MB-231/LR and MCF-7/LR cells. **(B)** IC_50_ value of lapatinib was detected in transfected MDA-MB-231/LR and MCF-7/LR cells using MTT assay. **(C)** Cell viability was analyzed in transfected MDA-MB-231/LR and MCF-7/LR cells by MTT assay. **(D)** Number of colonies was detected in transfected MDA-MB-231/LR and MCF-7/LR cells by cell colony formation assay. **(E)** Apoptosis rates were assessed in transfected MDA-MB-231/LR and MCF-7/LR cells by flow cytometry assay. **(F, G)** Capacities of migration and invasion were examined in transfected MDA-MB-231/LR and MCF-7/LR cells by transwell assay. **P < 0.01, ***P < 0.001, ****P < 0.0001 (ANOVA with Tukey’s tests), circ-MMP11 relative to GAPDH.

In view of the high expression of circ-MMP11 in LR-resistant breast cancer cells, we knocked down circ-MMP11 in MDA-MB-231/LR and MCF-7/LR cells. Results indicated that circ-MMP11 level was significantly downregulated in si-circ-MMP11-transfected MDA-MB-231/LR and MCF-7/LR cells versus in that in si-NC-transfected cells (**Figure 3A**). Subsequently, the loss-of-function experiments were applied to probe the effect of circ-MMP11 on the lapatinib resistance. IC_50_ determination presented that circ-MMP11 knockdown repressed the lapatinib resistance in MDA-MB-231/LR and MCF-7/LR cells (**Figure 3B**). Functionally, the results of MTT and colony formation assays suggested that the introduction of si-circ-MMP11 remarkably hindered cell viability and colony formation was remarkably hindered after the introduction of si-circ-MMP11 in MDA-MB-231/LR and MCF-7/LR cells (**Figures 3C, D**). Also, enhanced cell apoptosis was noted caused by the downregulation of circ-MMP11 in MDA-MB-231/LR and MCF-7/LR cells (**Figure 3E**). Meanwhile, transwell results indicated that circ-MMP11 deficiency prominently impaired the abilities of migration (**Figure 3F**) and invasion (**Figure 3G**) in MDA-MB-231/LR and MCF-7/LR cells. These mentioned results discovered that circ-MMP11 silencing sensitized MDA-MB-231/LR and MCF-7/LR cells to lapatinib.

### Circ-MMP11 Directly Interacted With miR-153-3p in LR Breast Cancer Cells

**Figure 4:**
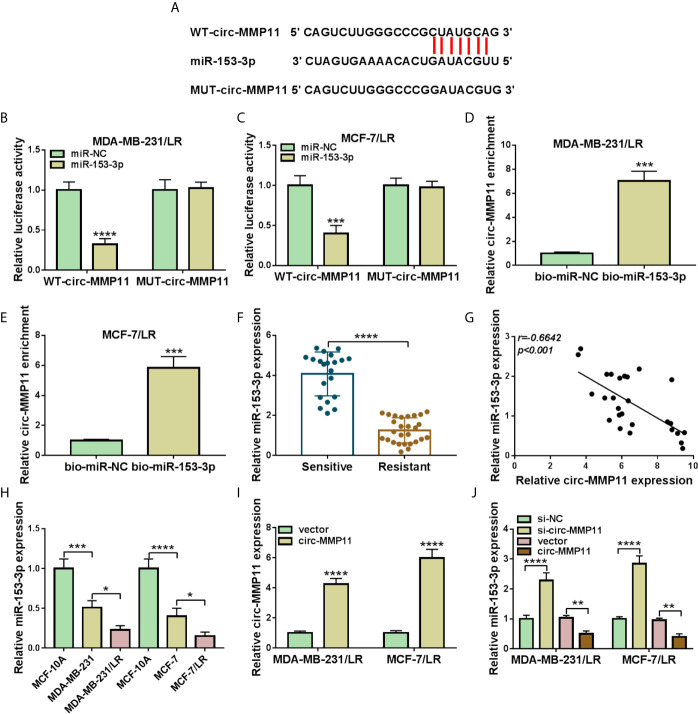
MiR-153-3p was a direct target of circ-MMP11 in LR breast cancer cells. **(A)** The binding between circ-MMP11 and miR-153-3p was predicated by using circinteractome software. **(B, C)** Dual-luciferase reporter assay was applied to analyze the prediction in MDA-MB-231/LR and MCF-7/LR cells. **(D, E)** RNA pull-down assay was conducted to detect the interaction between circ-MMP11 and miR-153-3p. **(F)** Relative miR-153-3p expression was detected in 21 chemo-sensitive tissues and 27 drug-resistant tissues. **(G)** Expression correlation between circ-MMP11 with miR-153-3p in drug-resistant tissues was analyzed by using Pearson correlation analysis. **(H)** Expression level of miR-153-3p was detected in MCF-10A, MDA-MB-231, MDA-MB-231/LR, MCF-7, MCF-7/LR cells. **(I)** Circ-MMP11 level was measured in MDA-MB-231/LR and MCF-7/LR cells transfected with vector and circ-MMP11. **(J)** MiR-153-3p level was assessed in MDA-MB-231/LR and MCF-7/LR cells transfected with si-NC, si-circ-MMP11, vector, and circ-MMP11. *P < 0.05, **P < 0.01, ***P < 0.001, ****P < 0.0001 (Student’s *t*-test or ANOVA with Tukey’s tests), circ-MMP11 relative to GAPDH, miR-153-3p relative to U6.

CircRNAs have been reported to perform the functional effect through interacting with specific miRNAs. Thus, we used the web-based tool circinteractome software to seek the underlying target miRNAs of circ-MMP11. As shown in **Figure 4A**, miR-153-3p was predicted to possess some complementary bases pairing with circ-MMP11. The following dual-luciferase reporter assay further verified the prediction. Data indicated that the luciferase activity in MDA-MB-231/LR and MCF-7/LR cells transfected with WT-circ-MMP11 and miR-153-3p mimic was markedly suppressed compared with that in cells transfected with WT-circ-MMP11 and miR-NC, whereas there was no remarkable effect in the cells with MUT-circ-MMP11 (**Figures 4B, C**). To verify the direct interaction between circ-MMP11 and miR-153-3p, RNA pull-down assay was performed in MDA-MB-231/LR and MCF-7/LR cells. As shown in **Figures 4D, E**, circ-MMP11 enrichment in the bio-miR-153-3p group was higher than in the bio-miR-NC group. Also, we found that compared to the drug-sensitive tissues, miR-153-3p level was obviously downregulated and inversely associated with circ-MMP11 level in drug-resistant tissues (**Figures 4F, G**). Synchronously, the trend of miR-153-3p expression in breast cancer cellular level was consistent with that in tissues (**Figure 4H**). Besides, we detected that the authentic effect of circ-MMP11 on miR-153-3p level in LR breast cancer cells. First, the overexpression efficiency of pcDNA-circ-MMP11 was measured and presented in MDA-MB-231/LR and MCF-7/LR cells (**Figure 4I**). Then, RT-qPCR assay suggested that the deficiency of circ-MMP11 increased the expression level of miR-153-3p, on the contrary, the overexpression of circ-MMP11 blocked miR-153-3p level in MDA-MB-231/LR and MCF-7/LR cells (**Figure 4J**). Taken together, these data suggested that circ-MMP11 interacted with miR-153-3p to repress its expression.

### Circ-MMP11 Silencing Decreased Lapatinib Resistance in LR Breast Cancer Cells by Modulating miR-153-3p

**Figure 5:**
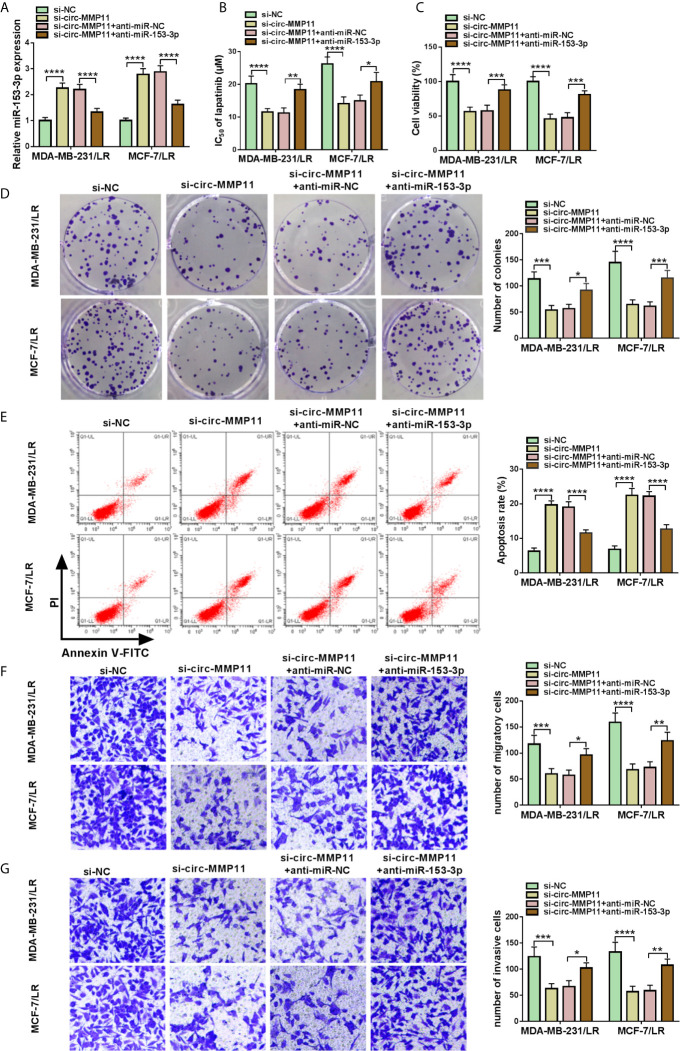
Downregulation of miR-153-3p mitigated circ-MMP11 silencing-mediated lapatinib sensitivity in LR breast cancer cells. MDA-MB-231/LR and MCF-7/LR cells were transfected with si-NC, si-circ-MMP11, si-circ-MMP11+anti-miR-NC, and si-circ-MMP11+ anti-miR-153-3p. **(A)** The expression level of miR-153-3p was measured in transfected MDA-MB-231/LR and MCF-7/LR cells. **(B)** IC_50_ value of lapatinib was detected by MTT assay in transfected MDA-MB-231/LR and MCF-7/LR cells. **(C, D)** Cell viability and clone number were determined by MTT and cell colony formation assays in transfected MDA-MB-231/LR and MCF-7/LR cells. **(E)** Apoptosis rate was evaluated by flow cytometry assay in transfected MDA-MB-231/LR and MCF-7/LR cells. **(F, G)** Abilities of migration and invasion were monitored by transwell assays in transfected MDA-MB-231/LR and MCF-7/LR cells. *P < 0.05, **P < 0.01, ***P < 0.001, ****P < 0.0001 (ANOVA with Tukey’s tests), miR-153-3p relative to U6.

Considering the regulatory effect of circ-MMP11 on miR-153-3p expression in MDA-MB-231/LR and MCF-7/LR cells, we further investigated whether the impact of circ-MMP11 on LR resistance was linked to the miR-153-3p. As presented in **Figure 5A**, the downregulation of circ-MMP11 distinctly intensified miR-153-3p expression level, which was counteracted by the introduction of anti-miR-153-3p in MDA-MB-231/LR and MCF-7/LR cells. Moreover, IC_50_ determination indicated that si-circ-MMP11 effectively blocked lapatinib resistance in MDA-MB-231/LR and MCF-7/LR cells, while the downregulation of miR-153-3p evidently abolished the effect (**Figure 5B**). Functional analysis suggested that miR-153-3p inhibitor could significantly ameliorate the suppressive effect of circ-MMP11 knockdown on cell viability and colony formation in MDA-MB-231/LR and MCF-7/LR cells (**Figures 5C, D**). In addition, circ-MMP11 deletion-mediated enhancement in cell apoptosis and decline in the abilities of migration and invasion were abrogated after co-transfection of anti-miR-153-3p in MDA-MB-231/LR and MCF-7/LR cells (**Figures 5E–G**). These mentioned results unraveled that circ-MMP11 knockdown improved lapatinib sensitivity through interacting with miR-153-3p in LR breast cancer cells.

### ANLN Worked as the Target of miR-153-3p in LR Breast Cancer Cells

**Figure 6:**
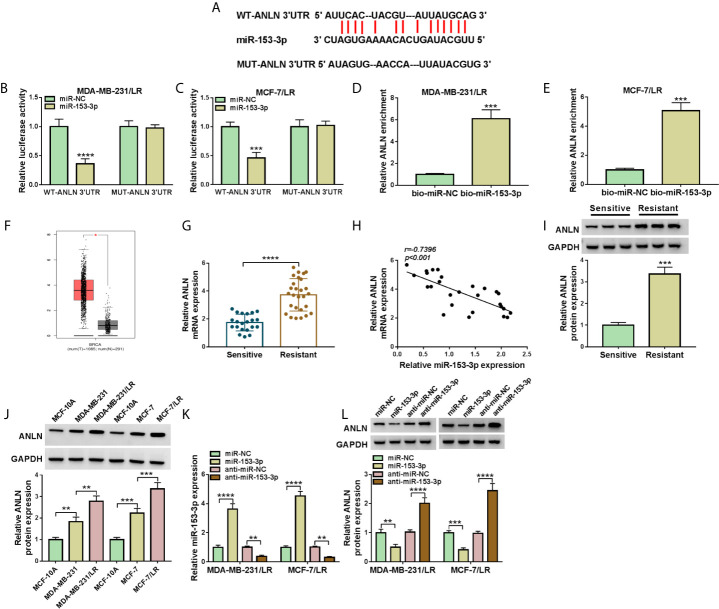
ANLN served as the target of miR-153-3p in LR breast cancer cells. **(A)** Wild-type or mutant miR-153-3p-binding sites in the 3’UTR sequences of ANLN were presented. **(B, C)** The effects of miR-153-3p upregulation on luciferase activity of WT-ANLN 3’UTR and MUT-ANLN 3’UTR reporters were analyzed in MDA-MB-231/LR and MCF-7/LR cells. **(D, E)** The interaction was confirmed by RNA pull-down assay. **(F)** GEPIA analysis showed that the expression of ANLN in BRCA tumor tissues (n = 1,085) and normal tissues (n = 291). **(G)** ANLN level was detected in the drug-sensitive group (n = 21) and the drug-resistant group (n = 27). **(H)** Pearson correlation analysis was applied to assess the expression association between miR-153-3p and ANLN in drug-resistant tissues. **(I, J)** ANLN protein level was tested in the drug-sensitive tissues, drug-resistant tissues, MCF-10A, MDA-MB-231, MDA-MB-231/LR, MCF-7, MCF-7/LR cells. **(K, L)** MiR-153-3p level and ANLN protein level were detected in MDA-MB-231/LR and MCF-7/LR cells transfected with miR-NC, miR-153-3p, anti-miR-NC, and anti-miR-153-3p, respectively. **P < 0.01, ***P < 0.001, ****P < 0.0001 (Student’s *t*-test or ANOVA with Tukey’s tests), miR-153-3p relative to U6, ANLN relative to GAPDH.

Then, in order to further explore the regulatory mechanism of miR-153-3p in breast cancer cells, bioinformatics software Starbase was applied to search the candidate target genes of miR-153-3p. As a result, we found that there were some binding sites between miR-153-3p and ANLN 3’UTR (**Figure 6A**). Consistent with the bioinformatics analysis, dual-luciferase reporter results suggested that miR-153-3p mimic could reduce the luciferase activity of WT-ANLN 3’UTR reporter but not that of MUT-ANLN 3’UTR reporter in MDA-MB-231/LR and MCF-7/LR cells (**Figures 6B, C**). And RNA pull-down assay suggested that the ANLN enrichment was significantly enhanced in the bio-miR-153-3p group compared with the bio-miR-NC group (**Figures 6D, E**). In addition, GEPIA analysis suggested that the expression level of ANLN was upregulated in 1,085 breast invasive carcinoma (BRCA) tissues when compared with 291 normal tissues (**Figure 6F**). Apart from that, negatively correlated with miR-153-3p expression level, ANLN was upregulated in the drug-resistant group (**Figures 6G, H**). Consistently, western blot analysis displayed that ANLN protein level was increased in the drug-resistant tissues and LR-resistant breast cancer cells versus their respective controls (**Figures 6I, J**). Subsequently, we further identify the influence of miR-153-3p expression on ANLN in LR-resistant breast cancer cells. The transfection efficiency of miR-153-3p mimic or miR-153-3p inhibitor was detected and exhibited in MDA-MB-231/LR and MCF-7/LR cells (**Figure 6K**). Then, we found that the upregulation of miR-153-3p could curb the protein level of ANLN, and the downregulation of miR-153-3p could augment ANLN protein level in MDA-MB-231/LR and MCF-7/LR cells (**Figure 6L**). All in all, miR-153-3p directly interacted with ANLN in LR breast cancer cells.

### MiR-153-3p Elevated Lapatinib Sensitivity by Targeting ANLN in LR Breast Cancer Cells

**Figure 7:**
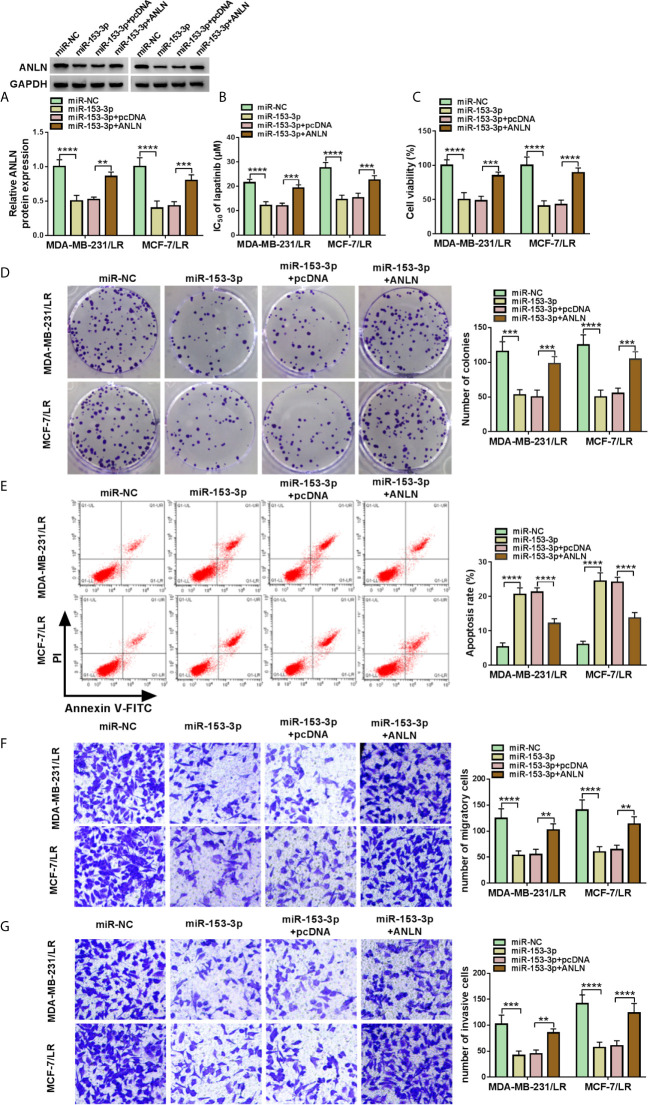
MiR-153-3p attenuated the lapatinib resistance by targeting ANLN in LR breast cancer cells. MDA-MB-231/LR and MCF-7/LR cells were transfected with miR-NC, miR-153-3p, miR-153-3p+pcDNA, and miR-153-3p+ANLN. **(A)** ANLN protein level was determined in transfected MDA-MB-231/LR and MCF-7/LR cells. **(B, C)** IC _50_ of LR and cell viability were measured in transfected MDA-MB-231/LR and MCF-7/LR cells. **(D, E)** Colony number and apoptosis rate were detected in transfected MDA-MB-231/LR and MCF-7/LR cells. **(F, G)** Migration and invasion were examined in transfected MDA-MB-231/LR and MCF-7/LR cells. **P < 0.01, ***P < 0.001, ****P < 0.0001 (ANOVA with Tukey’s tests), ANLN relative to GAPDH.

As mentioned above, miR-153-3p played an important role in lapatinib resistance in LR breast cancer cells. Meanwhile, ANLN acted as a latent target of miR-153-3p. Therefore, we further explored the relationship between miR-153-3p and ANLN in lapatinib resistance of LR breast cancer cells. As displayed in **Figure 7A**, the upregulation of miR-153-3p suppressed ANLN protein level in MDA-MB-231/LR and MCF-7/LR cells, whereas the re-introduction of pcDNA-ANLN evidently relieved the effects. Drug resistance assay showed that miR-153-3p mimic decreased lapatinib resistance in MDA-MB-231/LR and MCF-7/LR cells, which was obviously overturned due to the overexpression of ANLN (**Figure 7B**). What’s more, decreased cell viability and colony number caused by the upregulation of miR-153-3p was abolished by pcDNA-ANLN in MDA-MB-231/LR and MCF-7/LR cells (**Figures 7C, D**). Additional, enforced expression of ANLN effectively reversed miR-153-3p inhibitor-induced increase in cell apoptosis and repression in the abilities of migration and invasion in MDA-MB-231/LR and MCF-7/LR cells (**Figures 7E–G**). All of these data suggested that miR-153-3p could attenuate lapatinib resistance by regulating ANLN in LR breast cancer cells.

### ANLN Was Positively Regulated Through the circ-MMP11/miR-153-3p

**Figure 8:**
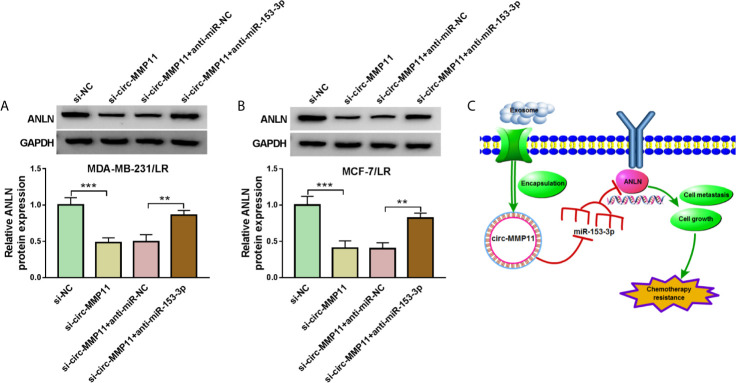
Circ-MMP11 could regulate ANLN expression by sponge miR-153-3p. **(A, B)** ANLN protein level was detected in MDA-MB-231/LR and MCF-7/LR cells transfected with si-NC, si-circ-MMP11, si-circ-MMP11 + anti-miR-NC, and si-circ-MMP11 + anti-miR-153-3p. **(C)** Exosomal circ-MMP11 could promote chemotherapy resistance by regulating cell growth and metastasis *via the* miR-153-3p/ANLN axis. **P < 0.01, ***P < 0.001 (ANOVA with Tukey’s tests), ANLN relative to GAPDH.

Based on the above results, we inferred that the regulatory role of circ-MMP11 could be mediated by the miR-153-3p/ANLN axis in LR breast cancer cells. To validate the speculation, rescue assays were carried out in MDA-MB-231/LR and MCF-7/LR cells. The results of western blot assays exhibited that the deficiency of circ-MMP11 repressed the protein level of ANLN; however, anti-miR-153-3p could significantly abolish the inhibitory impact of si-circ-MMP11 on ANLN expression in MDA-MB-231/LR and MCF-7/LR cells (**Figures 8A, B**). Also, we showed the exosomal circ-MMP11/miR-153-3p/ANLN axis in cell growth, metastasis, and chemotherapy resistance in LR-resistant breast cancer cells (**Figure 8C**). Besides, our data also suggested that exo-circ-MMP11-secreted by LR breast cancer cells accelerated lapatinib resistance, proliferation, migration, invasion of breast cancer cells by regulating the miR-153-3p/ANLN axis (**Figure S3**). In addition, the functional analysis suggested that the downregulation of ANLN partly reversed the suppressive role of exo-circ-MMP11 on cell growth and metastasis in breast cancer cells (**Figure S4**).

### Circ-MMP11 Knockdown Impeded Tumor Growth and Elevated Lapatinib Sensitivity *In Vivo*

**Figure 9:**
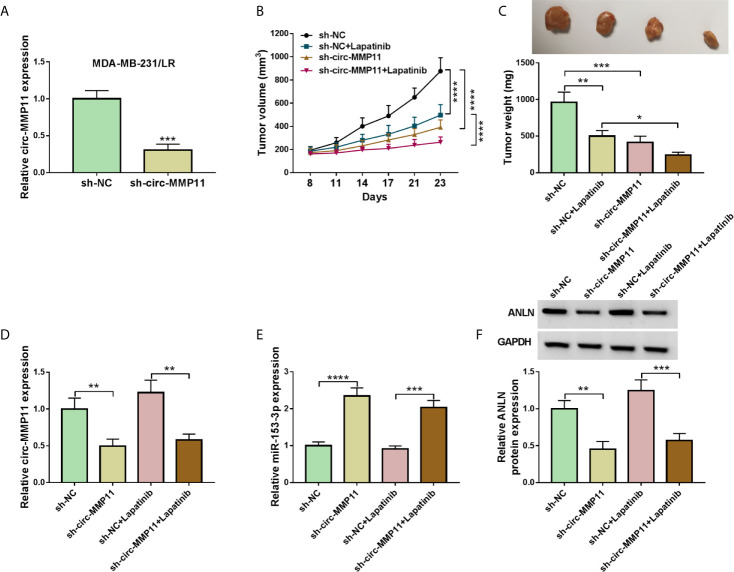
Circ-MMP11 deficiency suppressed tumor growth and improved lapatinib sensitivity in *vivo*. **(A)** RT-qPCR assay was used to determine circ-MMP11 expression in sh-circ-MMP11or sh-NC-introduced MDA-MB-231/LR cells. **(B, C)** Tumor volume and tumor weight were detected in xenografts. **(D)** The expression level of circ-MMP11 was examined in xenografts. **(E, F)** miR-153-3p and ANLN level were detected by RT-qPCR assay and western blot assay in this xenograft. *P < 0.05, **P < 0.01, ***P < 0.001, ****P < 0.0001 (Student’s *t*-test or ANOVA with Tukey’s tests), circ-MMP11 relative to GAPDH, miR-153-3p relative to U6, ANLN relative to GAPDH.

Furthermore, to identify the functional role of circ-MMP11 on lapatinib resistance *in vivo*, mice xenograft models of breast cancer were established. To begin with, our data suggested that circ-MMP11 expression was significantly decreased in sh-circ-MMP11-introduced MDA-MB-231/LR cells relative to cells with sh-NC (**Figure 9A**). After that, xenograft formation assay revealed that the tumor volume and weight were declined in the presence of circ-MMP11 downregulation or lapatinib treatment, hinting at the repression role of circ-MMP11 knockdown or lapatinib treatment on tumor growth (**Figures 9B, C**). Interestingly, we found that combined sh-circ-MMP11 and lapatinib showed a more distinct suppression on tumor growth. Also, our data proved that the expression levels of circ-MMP11 and ANLN were significantly decreased in tumor tissues derived from the sh-circ-MMP11 groups relative to the sh-NC groups (**Figures 9D, F**), while miR-153-3p expression presented an opposite trend in this xenograft (**Figure 9E**). In a word, circ-MMP11 knockdown improved lapatinib sensitivity of breast cancer *in vivo*.

## Discussion

Currently, lapatinib-based molecular targeted therapy has been considered particularly effective in the majority of cancer cases, but the lapatinib resistance is limiting the effectiveness of treatment in patients (20, 21). Furthermore, circRNAs have attracted wide attention to the important role in tumor progression and drug resistance (22). For example, hsa_circ_0063809 as an underlying biomarker, could confer paclitaxel resistance through binding to the miR-1252 in epithelial ovarian cancer (23). Analogously, circ-HIPK3 improved the gemcitabine resistance through promoting cell viability and metastasis in pancreatic cancer cells (24). However, the analysis of circRNAs and lapatinib resistance in breast cancer has not been reported. Besides, exosomes have attracted wide attention for the new means of intracellular communication (25, 26). Through selectively packaging, secreting, and transferring ncRNAs between cells, tumor-derived exosomes could participate in the numerous hallmarks of breast cancer, including proliferation, metastasis, and drug resistance (17, 27, 28). Of note, exosomal circRNAs have been a hot research area for their important role in various human cancers (29–31). As a new circRNA, circ-MMP11 has been presented the carcinogenesis by boosting cell proliferation and migration in breast cancer (12). In this work, circ-MMP11 was first identified to display the high expression in LR breast cancer tissues and cells, implying that circ-MMP11 might participate in the lapatinib resistance in breast cancer. Moreover, our data suggested that exosomal circ-MMP11 level was increased in LR breast cancer tissues. Importantly, we found that circ-MMP11 could be transferred by exosomes in breast cancer, suggesting the significance of circ-MMP11 in breast cancer cells.

Therefore, circ-MMP11 was chosen for in-depth investigations in breast cancer. In this paper, our data verified that circ-MMP11 knockdown could enhance the lapatinib sensitivity, hinder cell viability, colony number, migration, invasion, and induce apoptosis in LR breast cancer cells *in vitro*. Apart from that, the repression role of circ-MMP11 deficiency on tumor growth and lapatinib resistance of breast cancer cells was verified *in vivo*. In other words, we were first demonstrated that circ-MMP11 conferred lapatinib resistance in LR breast cancer cells *in vitro* and *in vivo.*

As widely believed, circRNAs predominantly located in the cytoplasm could exert biological function through acting as a ceRNA or sponge of microRNAs (miRNAs). The cytoplasmic expression of circ-MMP11 in breast cancer cells and LR breast cancer cells indicated the post-transcriptional regulatory mechanism of circ-MMP11 in breast cancer. Here, miR-153-3p as a target of circ-MMP11 was verified in LR breast cancer cells. Meanwhile, miR-153-3p has exhibited a vital role in breast cancer progression by inhibiting malignant behaviors and tumorigenesis (32). Also, it has confirmed the involvement of miR-153-3p and drug resistance in diverse human cancers (33, 34). The current work shows the low expression of miR-153-3p in LR breast cancer. Functionally, the downregulation of miR-153-3p partly reversed circ-MMP11 knockdown-mediated enhancement in lapatinib sensitivity, and decrease cell growth and metastasis of LR breast cancer cells. Several studies have stated that miRNAs could regulate tumor progression by interacting with mRNAs. In this paper, ANLN was verified to be a target of miR-153-3p. As an actin-binding protein, ANLN has been presented to be frequently upregulated in some human cancers (35–37), including breast cancer (38). Moreover, earlier kinds of literature have described that the ANLN expression could mediate drug-resistance of breast cancer cells to doxorubicin and anthracycline (39, 40). Here, our data proved that ANLN was over-expressed and negatively associated with a miR-153-3p level in LR breast cancer tissues. In addition, the overexpression of ANLN could abrogate the suppressive action of miR-153-3p on lapatinib resistance, cell growth, and metastasis of LR breast cancer cells.

Additionally, to further validate the regulatory role of circ-MMP11 could be mediated by the miR-153-3p/ANLN axis in LR breast cancer cells, rescue assays were performed. As expected, the downregulation of circ-MMP11 could repress ANLN expression level in LR breast cancer cells, and miR-153-3p knockdown partially eliminated the suppression effect of circ-MMP11 silencing on ANLN expression, further supporting the circ-MMP11/miR-153-3p/ANLN axis in LR breast cancer cells. In addition, our results also confirmed that the regulatory role of exo-circ-MMP11 on lapatinib resistance, cell growth, and metastasis could be mediated by the miR-153-3p/ANLN axis in LR breast cancer cells. Given that circ-MMP11 could be transferred by exosomes in breast cancer, and the effects of GW4869 on miR-153-3p and ANLN in this assay. We will continue to explore the regulatory role of exosomal circ-MMP11 on lapatinib resistance by regulating the miR-153-3p/ANLN axis in subsequent study.

## Conclusion

In summary, these results discovered that circ-MMP11 could be transferred by exosomes, and circ-MMP11 could elevate lapatinib resistance by regulating the miR-153-3p/ANLN axis in breast cancer cells. Our findings provided a better understanding of the mechanism of lapatinib resistance in breast cancer cells, hinting at a promising circRNA-targeted therapy for breast cancer.

## Data Availability Statement

The raw data supporting the conclusions of this article will be made available by the authors, without undue reservation.

## Ethics Statement

The studies involving human participants were reviewed and approved by The Affiliated Huaian No.1 People’s Hospital of Nanjing Medical University. The patients/participants provided their written informed consent to participate in this study. The animal study was reviewed and approved by The Affiliated Huaian No.1 People’s Hospital of Nanjing Medical University.

## Author Contributions

XW was responsible for drafting the manuscript. YR and RY contributed to the analysis and interpretation of data. LZ and RF contributed in the data collection. All authors contributed to the article and approved the submitted version.

## Conflict of Interest

The authors declare that the research was conducted in the absence of any commercial or financial relationships that could be construed as a potential conflict of interest.

## Supplementary Material

Supplementary Figure 1 Exosomal markers CD63 and CD9 were detected by western blot assay in supernatant and cell exosomes from MDA-MB-231 and MCF-7v cells.

Supplementary Figure 2 The effects of GW4869 on miR-153-3p and ANLN level, and proliferation ability in breast cancer cells. **(A)** miR-153-3p level was detected by RT-qPCR assay. **(B)** ANLN protein level was detected by western blot assay. **(C)** Cell viability was analyzed by MTT assay.

Supplementary Figure 3 Exosomal circ-MMP11 promoted lapatinib resistance, proliferation, migration, and invasion in lapatinib-sensitive breast cancer cells by interacting with miR-153-3p/ANLN axis. **(A–E)** MDA-MB-231 and MCF-7 cells were respectively treated with Exo-control or Exo-circ-MMP11-derived from MDA-MB-231/LR and MCF-7/LR cells. **(A)** IC_50_ value of lapatinib was analyzed in treated MDA-MB-231 and MCF-7 cells by MTT assay. **(B)** Cell viability of treated MDA-MB-231 and MCF-7 cells was measured by MTT assay. **(C)** Number of colonies of treated MDA-MB-231 and MCF-7 cells was examined by cell colony formation assay. **(D, E)** Migration and invasion capacities of treated MDA-MB-231 and MCF-7 cells were tested by Transwell assay. **(F)** ANLN protein level was detected in MDA-MB-231 and MCF-7 cells treated with PBS, exo-circ-MMP11, exo-circ-MMP11+miR-NC, exo-circ-MMP11+miR-153-3p by western blot assay. ***P <* 0.01, ****P <* 0.001, *****P <* 0.0001. (ANOVA with Tukey’s tests), ANLN relative to GAPDH.

Supplementary Figure 4 The downregulation of ANLN could abolish the effects of exo-circ-MMP11 on lapatinib resistance, proliferation, migration, and invasion in lapatinib-sensitive breast cancer cells. MDA-MB-231/LR and MCF-7/LR cells were treated with Exo-control or, Exo-circ-MMP11, Exo-circ-MMP11+si-NC, and Exo-circ-MMP11+si-ANLN. **(A)** ANLN protein level was detected in treated MDA-MB-231/LR and MCF-7/LR cells by western blot assay. **(B)** IC_50_ value of lapatinib was detected in treated MDA-MB-231/LR and MCF-7/LR cells using MTT assay. **(C)** Cell viability was measured in treated MDA-MB-231/LR and MCF-7/LR cells by MTT assay. **(D)** The number of colonies was examined in treated MDA-MB-231/LR and MCF-7/LR cells by cell colony formation assay. **(E, F)** Capacities of migration and invasion were tested in treated MDA-MB-231/LR and MCF-7/LR cells by Transwell assay. ***P <*0.01, ****P < * 0.001, *****P <* 0.0001. (ANOVA with Tukey’s tests), ANLN relative to GAPDH.

## References

[1] Rebholz WN Cash E Zimmaro LA Bayley-Veloso R Phillips K Siwik C. Distress and Quality of Life in an Ethnically Diverse Sample Awaiting Breast Cancer Surgery. J Health Psychol (2018) 23:1438–51. 10.1177/1359105316659916 27466289

[2] Siegel RL Miller KD. Cancer Statistics. CA Cancer J Clin (2020) 2020:70:7–30. 10.3322/caac.21590 31912902

[3] Chan A. Lapatinib - Overview and Current Role in Metastatic Breast Cancer. Cancer Res Treat (2006) 38:198–200. 10.4143/crt.2006.38.4.198 19771242 PMC2741644

[4] Rusnak DW Lackey K Affleck K Wood ER Alligood KJ Rhodes N. The Effects of the Novel, Reversible Epidermal Growth Factor Receptor/ErbB-2 Tyrosine Kinase Inhibitor, GW2016, on the Growth of Human Normal and Tumor-Derived Cell Lines *In Vitro* and *In Vivo* . Mol Cancer Ther (2001) 1:85–94. 10.1097/00008390-200112000-00011 12467226

[5] Campone M Juin P André F Bachelot T. Resistance to HER2 Inhibitors: Is Addition Better Than Substitution? Rationale for the Hypothetical Concept of Drug Sedimentation. Crit Rev Oncol Hematol (2011) 78:195–205. 10.1016/j.critrevonc.2010.04.012 20684884

[6] Wang L Zhang Q Zhang J Sun S Guo H Jia Z. PI3K Pathway Activation Results in Low Efficacy of Both Trastuzumab and Lapatinib. BMC Cancer (2011) 11:248. 10.1186/1471-2407-11-248 21676217 PMC3141770

[7] Liu L Greger J Shi H Liu Y Greshock J Annan R. Novel Mechanism of Lapatinib Resistance in HER2-positive Breast Tumor Cells: Activation of AXL. Cancer Res (2009) 69:6871–8. 10.1158/0008-5472.CAN-08-4490 19671800

[8] Kristensen LS Andersen MS Stagsted LVW Ebbesen KK Hansen TB. The Biogenesis, Biology and Characterization of Circular RNAs. Nat Rev Genet (2019) 20:675–91. 10.1038/s41576-019-0158-7 31395983

[9] Gao D Zhang X Liu B Meng D Fang K Guo Z. Screening Circular RNA Related to Chemotherapeutic Resistance in Breast Cancer. Epigenomics (2017) 9:1175–88. 10.2217/epi-2017-0055 28803498

[10] Zang H Li Y Zhang X Huang G. Circ-RNF111 Contributes to Paclitaxel Resistance in Breast Cancer by Elevating E2F3 Expression *Via* miR-140-5p. Thorac Cancer (2020) 11:1891–903. 10.1111/1759-7714.13475 PMC7327676 32445273

[11] Sang Y Chen B Song X Li Y Liang Y Han D. CircRNA_0025202 Regulates Tamoxifen Sensitivity and Tumor Progression *Via* Regulating the miR-182-5p/FOXO3a Axis in Breast Cancer. Mol Ther (2019) 27:1638–52. 10.1016/j.ymthe.2019.05.011 PMC6731174 31153828

[12] Li Z Chen Z Feng Y Hu G Jiang Y. CircMMP11 Acts as a ce-circRNA in Breast Cancer Progression by Regulating miR-1204. Am J Transl Res (2020) 12:2585–99. PMC7344057 32655792

[13] Théry C Zitvogel L Amigorena S. Exosomes: Composition, Biogenesis and Function. Nat Rev Immunol (2002) 2:569–79. 10.1038/nri855 12154376

[14] Mashouri L Yousefi H Aref AR Ahadi AM Molaei F Alahari SK. Exosomes: Composition, Biogenesis, and Mechanisms in Cancer Metastasis and Drug Resistance. Mol Cancer (2019) 18:75. 10.1186/s12943-019-0991-5 30940145 PMC6444571

[15] Fanale D Taverna S Russo A Bazan V. Circular RNA in Exosomes. Adv Exp Med Biol (2018) 1087:109–17. 10.1007/978-981-13-1426-1_9 30259361

[16] Luo Y Gui R. Circulating Exosomal circFoxp1 Confers Cisplatin Resistance in Epithelial Ovarian Cancer Cells. Gynecol Oncol (2020) 31:e75. 10.3802/jgo.2020.31.e75 PMC7440976 32808501

[17] Hu K Liu X Li Y Li Q Xu Y Zeng W. Exosomes Mediated Transfer of Circ_UBE2D2 Enhances the Resistance of Breast Cancer to Tamoxifen by Binding to MiR-200a-3p. Med Sci Monit (2020) 26:e922253. 10.12659/MSM.922253 32756532 PMC7431386

[18] Corcoran C Rani S Breslin S Gogarty M Ghobrial IM Crown J. miR-630 Targets IGF1R to Regulate Response to HER-targeting Drugs and Overall Cancer Cell Progression in HER2 Over-Expressing Breast Cancer. Mol Cancer (2014) 13:71. 10.1186/1476-4598-13-71 24655723 PMC4234346

[19] Piao HY Guo S. Exosomal Long Non-Coding RNA CEBPA-AS1 Inhibits Tumor Apoptosis and Functions as a Non-Invasive Biomarker for Diagnosis of Gastric Cancer. Onco Targets Ther (2020) 13:1365–74. 10.2147/OTT.S238706 PMC7034294 32110038

[20] D’Amato V Raimondo L Formisano L Giuliano M De Placido S Rosa R. Mechanisms of Lapatinib Resistance in HER2-Driven Breast Cancer. Cancer Treat Rev (2015) 41:877–83. 10.1016/j.ctrv.2015.08.001 26276735

[21] Ruprecht B Zaal EA Zecha J Wu W Berkers CR Kuster B. Lapatinib Resistance in Breast Cancer Cells Is Accompanied by Phosphorylation-Mediated Reprogramming of Glycolysis. Cancer Res (2017) 77:1842–53. 10.1158/0008-5472.CAN-16-2976 28209619

[22] Geng X Jia Y Zhang Y Shi L Li Q Zang A. Circular RNA: Biogenesis, Degradation, Functions and Potential Roles in Mediating Resistance to Anticarcinogens. Epigenomics (2020) 12:267–83. 10.2217/epi-2019-0295 31808351

[23] Zhang S Cheng J Quan C Wen H Feng Z Hu Q. circCELSR1 (hsa_circ_0063809) Contributes to Paclitaxel Resistance of Ovarian Cancer Cells by Regulating Foxr2 Expression *Via* miR-1252. Mol Ther Nucleic Acids (2020) 19:718–30. 10.1016/j.omtn.2019.12.005 PMC6965731 31945729

[24] Liu Y Xia L Dong L Wang J Xiao Q Yu X. CircHIPK3 Promotes Gemcitabine (GEM) Resistance in Pancreatic Cancer Cells by Sponging miR-330-5p and Targets RASSF1. Cancer Manag Res (2020) 12:921–9. 10.2147/CMAR.S239326 PMC7023912 32104074

[25] Couto N Caja S Maia J Strano Moraes MC Costa-Silva B. Exosomes as Emerging Players in Cancer Biology. Biochimie (2018) 155:2–10. 10.1016/j.biochi.2018.03.006 29555374

[26] Li I Nabet BY. Exosomes in the Tumor Microenvironment as Mediators of Cancer Therapy Resistance. Mol Cancer (2019) 18:32. 10.1186/s12943-019-0975-5 30823926 PMC6397467

[27] Li XJ Ren ZJ Tang JH Yu Q. Exosomal MicroRNA MiR-1246 Promotes Cell Proliferation, Invasion and Drug Resistance by Targeting CCNG2 in Breast Cancer. Cell Physiol Biochem (2017) 44:1741–8. 10.1159/000485780 29216623

[28] Dong H Wang W Chen R Zhang Y Zou K Ye M. Exosome-Mediated Transfer of lncRNA−SNHG14 Promotes Trastuzumab Chemoresistance in Breast Cancer. Int J Oncol (2018) 53:1013–26. 10.3892/ijo.2018.4467 PMC6065402 30015837

[29] Li Z Yanfang W Li J Jiang P Peng T Chen K. Tumor-Released Exosomal Circular RNA PDE8A Promotes Invasive Growth *Via* the miR-338/MACC1/MET Pathway in Pancreatic Cancer. Cancer Lett (2018) 432:237–50. 10.1016/j.canlet.2018.04.035 29709702

[30] Lai Z Wei T Li Q Wang X Zhang Y Zhang S. Exosomal CircFBLIM1 Promotes Hepatocellular Carcinoma Progression and Glycolysis by Regulating the miR-338/LRP6 Axis. Cancer Biother Radiopharm (2020). 10.1089/cbr.2020.3564 32907351

[31] Han C Wang S Wang H Zhang J. Exosomal Circ-HIPK3 Facilitates Tumor Progression and Temozolomide Resistance by Regulating miR-421/ZIC5 Axis in Glioma. Cancer Biother Radiopharm (2020). 10.1089/cbr.2019.3492 32644821

[32] Yu L Xu Q Yu W Duan J Dai G. LncRNA Cancer Susceptibility Candidate 15 Accelerates the Breast Cancer Cells Progression *Via* miR-153-3p/KLF5 Positive Feedback Loop. Biochem Biophys Res Commun (2018) 506:819–25. 10.1016/j.bbrc.2018.10.131 30389133

[33] Zuo J Zhao M Fan Z Liu B Wang Y Li Y. MicroRNA-153-3p Regulates Cell Proliferation and Cisplatin Resistance *Via* Nrf-2 in Esophageal Squamous Cell Carcinoma. Thorac Cancer (2020) 11:738–47. 10.1111/1759-7714.13326 PMC7049518 32012470

[34] Joo LJS Weiss J Gill AJ Clifton-Bligh R Brahmbhatt H MacDiarmid JA. Ret Kinase-Regulated MicroRNA-153-3p Improves Therapeutic Efficacy in Medullary Thyroid Carcinoma. Thyroid (2019) 29:830–44. 10.1089/thy.2018.0525 30929576

[35] Wang A Dai H Gong Y Zhang C Shu J Luo Y. ANLN-Induced EZH2 Upregulation Promotes Pancreatic Cancer Progression by Mediating miR-218-5p/LASP1 Signaling Axis. J Exp Clin Cancer Res (2019) 38:347. 10.1186/s13046-019-1340-7 31395079 PMC6686567

[36] Xu J Zheng H Yuan S Zhou B Zhao W Pan Y. Overexpression of ANLN in Lung Adenocarcinoma Is Associated With Metastasis. Thorac Cancer (2019) 10:1702–9. 10.1111/1759-7714.13135 PMC6669805 31268619

[37] Zeng S Yu X Ma C Song R Zhang Z Zi X. Transcriptome Sequencing Identifies ANLN as a Promising Prognostic Biomarker in Bladder Urothelial Carcinoma. Sci Rep (2017) 7:3151. 10.1038/s41598-017-02990-9 28600503 PMC5466664

[38] Zhou W Wang Z Shen N Pi W Jiang W Huang J. Knockdown of ANLN by Lentivirus Inhibits Cell Growth and Migration in Human Breast Cancer. Mol Cell Biochem (2015) 398:11–9. 10.1007/s11010-014-2200-6 25223638

[39] Zhang M Wang F Xiang Z Huang T Zhou WB. LncRNA XIST Promotes Chemoresistance of Breast Cancer Cells to Doxorubicin by Sponging miR-200c-3p to Upregulate ANLN. Clin Exp Pharmacol Physiol (2020) 47:1464–72. 10.1111/1440-1681.13307 32198770

[40] Wang Z Chen J Zhong MZ Huang J Hu YP Feng DY. Overexpression of ANLN Contributed to Poor Prognosis of Anthracycline-Based Chemotherapy in Breast Cancer Patients. Cancer Chemother Pharmacol (2017) 79:535–43. 10.1007/s00280-017-3248-2 28243684

