# Supplementary figures and images for: Circular RNA circ-MMP11 Contributes to Lapatinib Resistance of Breast Cancer Cells by Regulating the miR-153-3p/ANLN Axis
Source: Front Oncol. 2021 Jul 6;11:639961. doi: 10.3389/fonc.2021.639961 (PMC8290203; doi:10.3389/fonc.2021.639961)

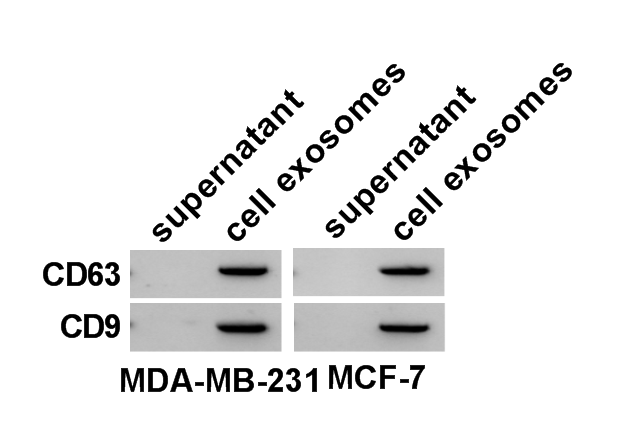

Supplement: Supplementary Figure 1 — Exosomal markers CD63 and CD9 were detected by western blot assay in supernatant and cell exosomes from MDA-MB-231 and MCF-7v cells. [file Image_1.tif]

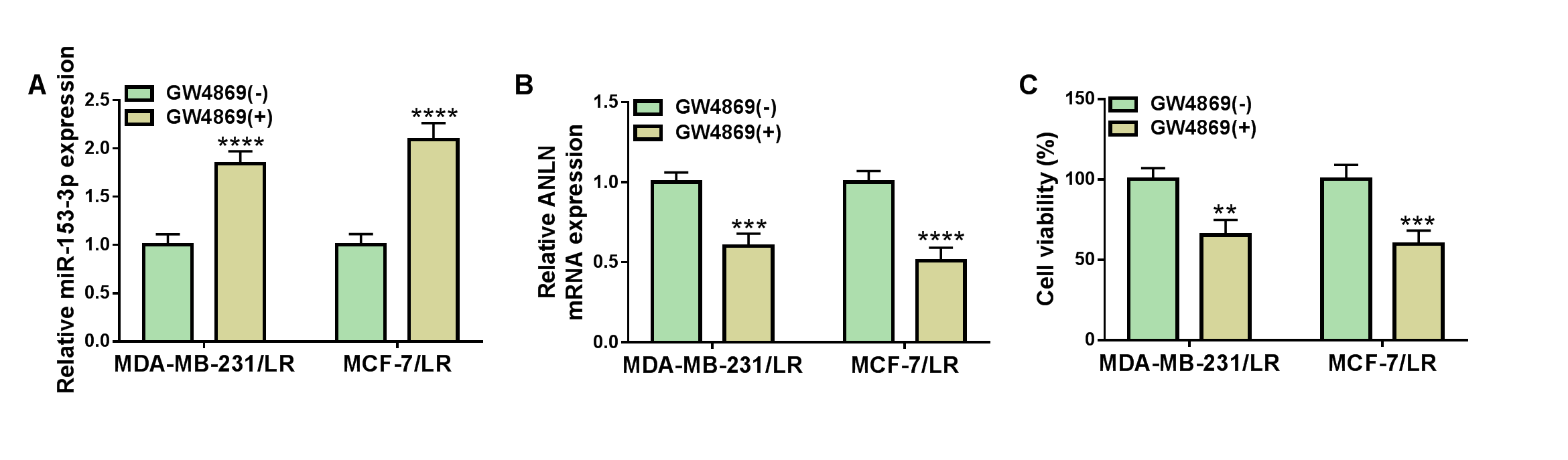

Supplement: Supplementary Figure 2 — The effects of GW4869 on miR-153-3p and ANLN level, and proliferation ability in breast cancer cells. (A) miR-153-3p level was detected by RT-qPCR assay. (B) ANLN protein level was detected by western blot assay. (C) Cell viability was analyzed by MTT assay. [file Image_2.tif]

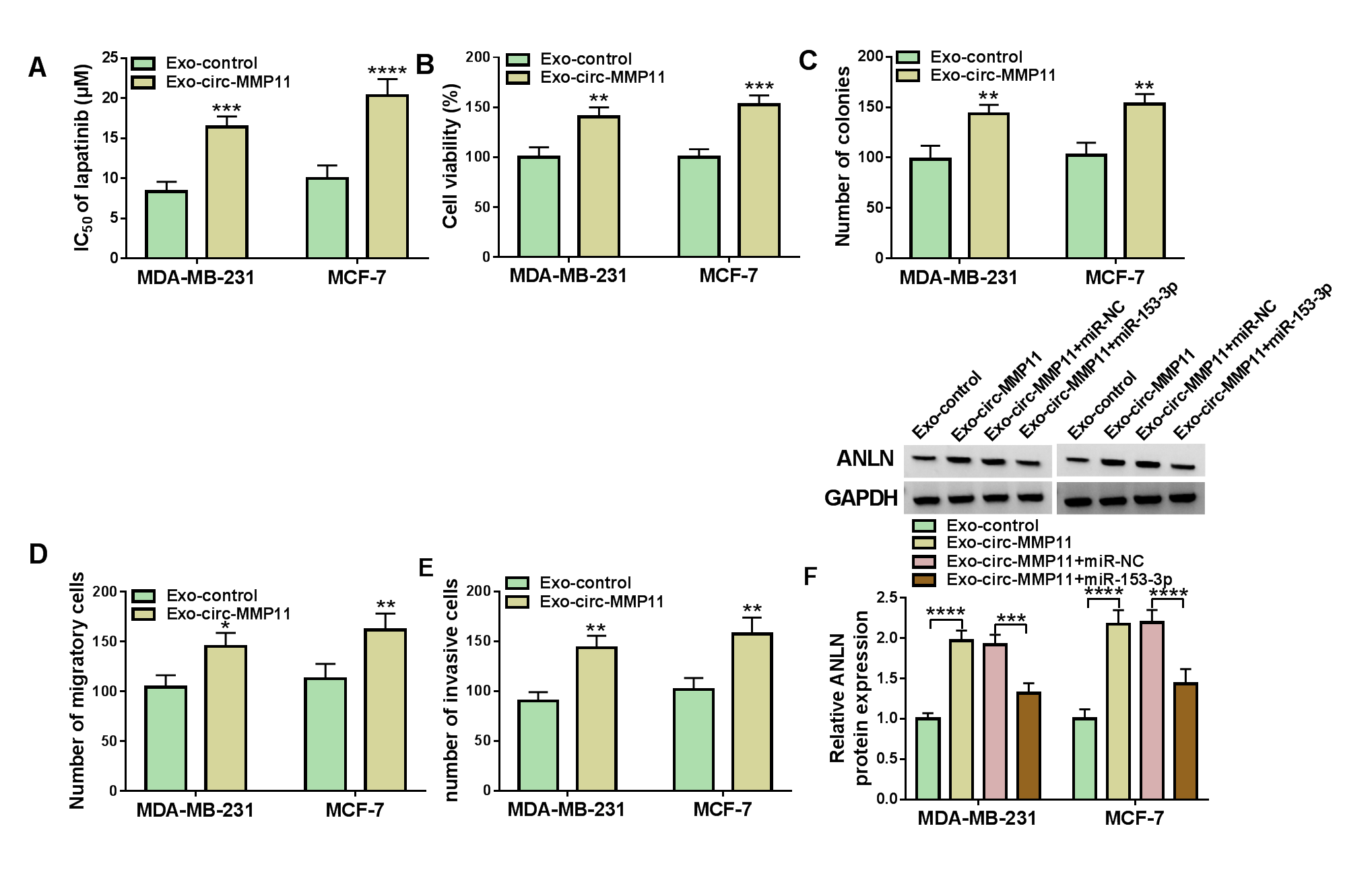

Supplement: Supplementary Figure 3 — Exosomal circ-MMP11 promoted lapatinib resistance, proliferation, migration, and invasion in lapatinib-sensitive breast cancer cells by interacting with miR-153-3p/ANLN axis. (A–E) MDA-MB-231 and MCF-7 cells were respectively treated with Exo-control or Exo-circ-MMP11-derived from MDA-MB-231/LR and MCF-7/LR cells. (A) IC50 value of lapatinib was analyzed in treated MDA-MB-231 and MCF-7 cells by MTT assay. (B) Cell viability of treated MDA-MB-231 and MCF-7 cells was measured by MTT assay. (C) Number of colonies of treated MDA-MB-231 and MCF-7 cells was examined by cell colony formation assay. (D, E) Migration and invasion capacities of treated MDA-MB-231 and MCF-7 cells were tested by Transwell assay. (F) ANLN protein level was detected in MDA-MB-231 and MCF-7 cells treated with PBS, exo-circ-MMP11, exo-circ-MMP11+miR-NC, exo-circ-MMP11+miR-153-3p by western blot assay. **P < 0.01, ***P < 0.001, ****P < 0.0001. (ANOVA with Tukey’s tests), ANLN relative to GAPDH. [file Image_3.tif]

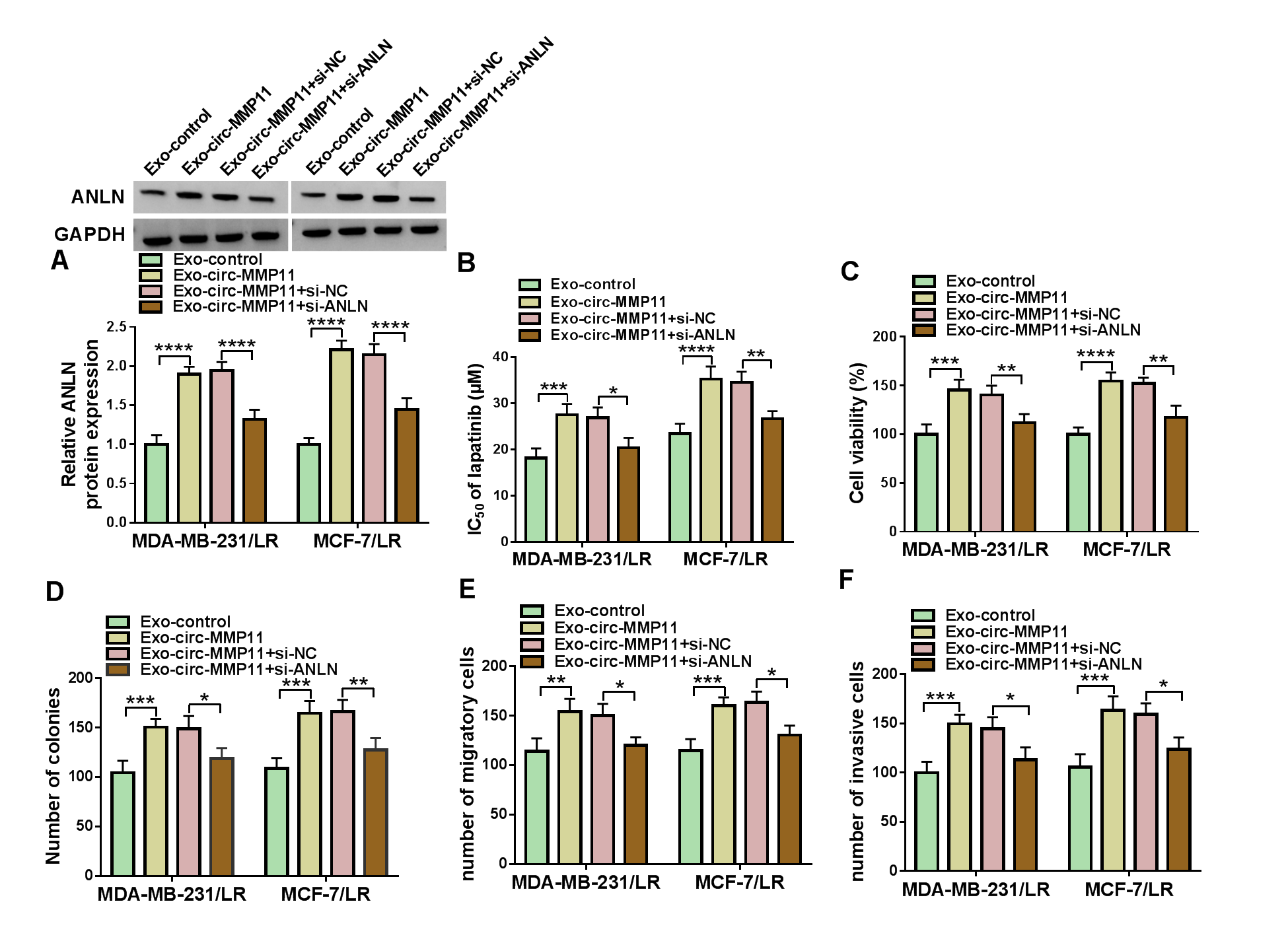

Supplement: Supplementary Figure 4 — The downregulation of ANLN could abolish the effects of exo-circ-MMP11 on lapatinib resistance, proliferation, migration, and invasion in lapatinib-sensitive breast cancer cells. MDA-MB-231/LR and MCF-7/LR cells were treated with Exo-control or, Exo-circ-MMP11, Exo-circ-MMP11+si-NC, and Exo-circ-MMP11+si-ANLN. (A) ANLN protein level was detected in treated MDA-MB-231/LR and MCF-7/LR cells by western blot assay. (B) IC50 value of lapatinib was detected in treated MDA-MB-231/LR and MCF-7/LR cells using MTT assay. (C) Cell viability was measured in treated MDA-MB-231/LR and MCF-7/LR cells by MTT assay. (D) The number of colonies was examined in treated MDA-MB-231/LR and MCF-7/LR cells by cell colony formation assay. (E, F) Capacities of migration and invasion were tested in treated MDA-MB-231/LR and MCF-7/LR cells by Transwell assay. **P <0.01, ***P < 0.001, ****P < 0.0001. (ANOVA with Tukey’s tests), ANLN relative to GAPDH. [file Image_4.tif]
